# Clinical Characteristics and Long-Term Outcome of Headaches Associated With Moyamoya Disease in the Chinese Population—A Cohort Study

**DOI:** 10.3389/fneur.2020.605636

**Published:** 2020-11-26

**Authors:** Bin Gao, Kaijiang Kang, Jia Zhang, Dong Zhang, Xingquan Zhao

**Affiliations:** ^1^Department of Neurology, Beijing Tiantan Hospital, Capital Medical University, Beijing, China; ^2^China National Clinical Research Center for Neurological Diseases, Beijing, China; ^3^Center of Stroke, Beijing Institute for Brain Disorders, Beijing, China; ^4^Beijing Key Laboratory of Translational Medicine for Cerebrovascular Disease, Beijing, China; ^5^Department of Neurosurgery, Beijing Tiantan Hospital, Capital Medical University, Beijing, China

**Keywords:** headaches associated with Moyamoya disease, HAMD, headache, Moyamoya disease, migraine-like, tension type-like headache, bypass intervention, placebo effect

## Abstract

**Background:** Headache associated with Moyamoya disease (HAMD) in the Chinese population is not well-described. The long-term outcome of surgical revascularization and natural course of HAMD has not been disclosed either.

**Methods:** A headache screening questionnaire in China based on the ICHD2 and a face-to-face interview performed by an experienced neurologist were used to investigate headache characteristics and frequency and pain intensity in the 3 months before admission, and a telephone interview was used for the follow-up of a large cohort of 119 Chinese patients with HAMD.

**Results:** Headache intensity was rated as scores of 5.9 ± 2.0 on a visual analog scale (VAS), ranging from 0 to 10, in the 3 months before admission. Forty-six patients (38.6%) were categorized as having migraine-like headaches, 29 patients (24.3%) were categorized as having tension type-like headaches, and 44 patients (36.9%) had a combination of both. The majority of patients had migraine-like headaches (*n* = 34, 73.9%) with a migrainous aura. Both the frequency and intensity of the headache improved significantly in patients treated with surgical revascularization (*n* = 96, 80.7%) or the conservative treatment (*n* = 23, 19.3%) in a long-term follow-up.

**Conclusion:** HAMD frequently presented with a migraine-like headache (75.5% in total). A tension type headache was present in 60.9% of patients. The symptom of dizziness is common in patients with HAMD (60.5%), and 19 of them (26.4%) met the diagnose of vestibular migraine. Both intensity and frequency of HAMD show a trend of spontaneous remission in a long-term follow-up, and there is no difference in long-term outcomes of HAMD between surgical revascularization and conservative treatment, which indicates that the effect of bypass intervention on HAMD may be a placebo effect.

## Introduction

Moyamoya disease (MMD), named first by Suzuki, is characterized by progressive steno-occlusion at the terminal portion of the internal carotid artery (ICA) accompanied by abnormal vascular networks (1–3). Headache is a common clinical manifestation in patients with MMD, but this is commonly underestimated in its clinical management (1, 4, 5). Several existing studies confirmed the high prevalence of headache in MMD, but the effect of surgical revascularization on headaches associated with MMD remains controversial (4–9). The precise quantifiable characteristics of HAMD and its pathology in Chinese patients remain unclear. We aimed to evaluate systematic headache symptoms, the effect of revascularization surgery, as well as the natural course of HAMD in a larger cohort of Chinese patients.

## Clinical Materials and Method

This study was approved by the Beijing Tiantan Hospital Institutional Review Board, and informed written consent were offered by all patients. We enrolled consecutively 119 patients from the inpatient department from January 2010 to September 2019 who met the inclusion criteria, including being diagnosed with MMD according to the published guidelines by the Research Committee on MMD (spontaneous occlusion of the circle of Willis) of Japan in 1997 (10), and headaches occurred at least once a month and lasted for at least 3 months before enrollment.

All participants received a questionnaire for screening headache in the Chinese population based on the diagnostic criteria of the Headache Classification Committee of the International Headache Society (IHS), The International Classification of Headache Disorders, 2nd edition(ICHD-2) (11), and a face-to-face interview performed by an experienced neurologist when they were enrolled. Furthermore, several questions were included to investigate symptoms/events (e.g., stroke and dizziness) related to HAMD. Those patients were also asked to record the frequency and intensity of headaches in the 3 months before enrollment. After a long-term telephone follow-up of those patients, the frequency and intensity of headaches in the past 3 months were recorded as well.

### Statistics

Data were presented descriptively. Group comparisons were performed using an ANOVA or *t*-test, whichever was most appropriate. Kruskal–Wallis tests were used to explore differences between headaches in those patients in whom headaches improved after surgery. Statistical analysis was completed with IBM SPSS version 23.0 (SPSS, Inc., Chicago, IL, United States). The level of significance was set to *p* < 0.05.

## Results

### Baseline Profiles of Study Patients

The study patients were 119 Chinese HAMD patients (male: female = 1:2.1). The mean follow-up time was 4.9 years (median: 5.0 years; SD: 2.3 years; range: 0.5–10 years). A unilateral variant of MMD (with assumed idiopathic etiology) was diagnosed in seven patients (5.8%), while the bilateral idiopathic disease was discovered in 112 patients (94.2%). Four patients (3.3%) were found to have an intracranial aneurysm as determined by digital subtraction angiography (DSA). The mean age of becoming a member of the study was 34.4 years (median: 37 years; SD: 15.5 years; range: 5–71 years). The mean follow-up time was 4.9 years (median: 5 years; SD: 2.3 years; range: 0.5–10 years). The median duration of headache before admission was 5.5 years (median: 2 years; SD: 7.5 years; range: 0.25–40 years). Ninety-eight patients (82.4%) had histories of stroke; among them, 63.0% had experienced a transient ischemic attack, 13.4% had a cerebral infarction, and 24.3% had a history of intracranial hemorrhage or subarachnoid hemorrhage.

### Headache

**Figure 1:**
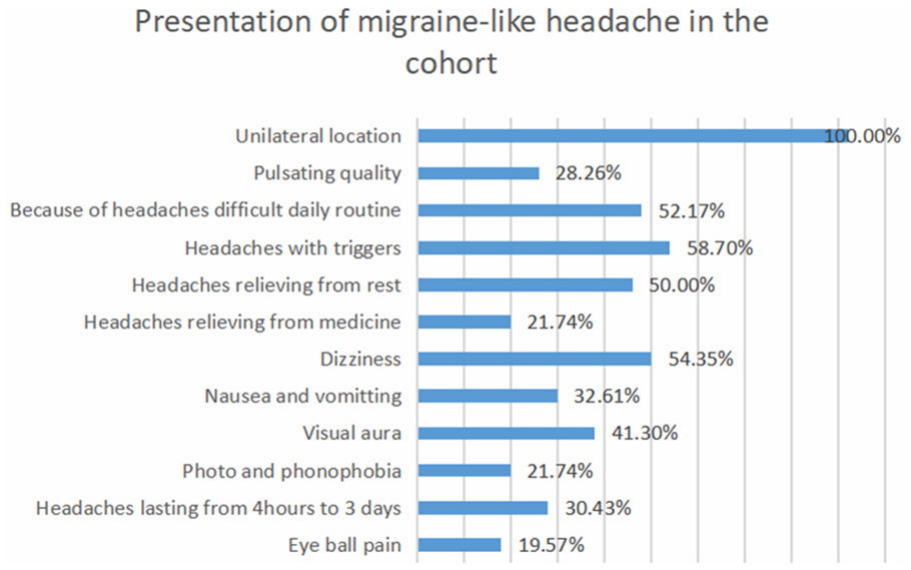
Presentation of migraine-like headache in the cohort.

**Figure 2:**
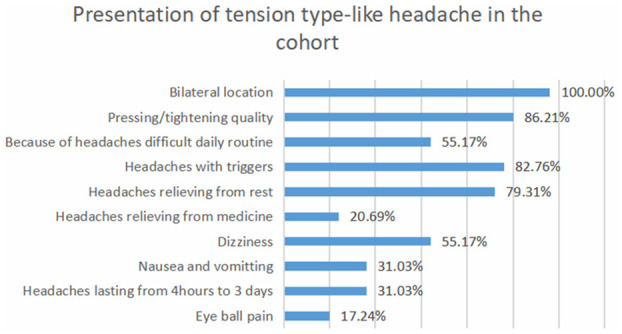
Presentation of tension-type-like headache in the cohort.

According to ICHD-2, 46 patients (38.6%) fulfilled the diagnostic criteria for migraine-like headaches. Thirty four of them (73.9%) fulfilled the diagnostic criteria for migraine with aura. Auras were visual in 19 patients (41.3%) and sensory in 18 patients (39.1%), and they involved motor weakness in 15 patients (32.6%) and speech disturbances in 13 patients (28.2%). Tension-type headache was reported in 29 patients (24.3%), and a combination of migraine-like and tension-type-like headache was reported in 44 patients (36.9%; Figures 1, 2 and Tables 1, 2).

**Table 1:** Baseline profiles of all patients in a large cohort of Chinese patients with HAMD.

**Total** ***N*** **=** **119**	
Aged >17y on admission (*n* %)	94 (79.0%)
Sex (female, *n* %)	81 (68.1%)
Follow-up time (years, mean ± SD)	4.949 ± 2.272
Median, range	5.000, 0.50–10.00
Duration of headaches before admission (years, mean ± SD)	5.484 ± 7.477
Median, range	2.000, 0.25–40.00
Prehistory of stroke (yes, *n* %)	98 (82.4%)
TIA (*n* %)	75 (63.0%)
Cerebral infarction (*n* %)	16 (13.4%)
ICH or SAH (*n* %)	29 (24.3%)
**Symptoms of headaches**	
With dizziness (*n* %)	72 (60.5%)
Headaches with triggers (*n* %)	85 (71.4%)
Because of headaches difficult daily routine (*n* %)	67 (56.3%)
**Imaging findings**	
Bilateral moyamoya angiopathy (*n* %)	112 (94.1%)
With intracranial aneurysm (*n* %)	4 (3.3%)
**Subtypes of headache**	
Migraine-like headache (*n* %)	46 (38.6%)
Tension type-like headache (*n* %)	29 (24.3%)
Migraine + TTH (*n* %)	44 (36.9%)
**Intervention**	
Surgical revascularization (*n* %)	96 (80.6%)
Conservative treatment (*n* %)	23 (19.4%)

**Table 2:** Frequency (days) and intensity (visual analog scale) of different subtypes of headaches in a large cohort of Chinese patients with HAMD when enrolled.

	**Migraine(M)**	**TTH**	**M+TTH**	***P*-value**	**95%CI**
**Total** ***n*** **=** **119**					
Headache(HA) frequency	13.89 ± 12.42 46 8.1–30	17.90 ± 12.00 29 15.1–30	17.77 ± 12.79 44 23.1–30	0.249	14.03–18.57
Headache(HA) intensity	5.70 ± 2.13 5.1–10	5.21 ± 1.99 5.2–9	6.44 ± 1.78 7.3–10	0.028	5.49–6.22

In our study, headaches make the daily routine more difficult in 67 patients (56.3%). A total of 85 patients (71.4%) had obvious triggers of headache, including emotional agitation, fatigue, crying, exercise, weather changes, and eating spicy, hot, or cold food. Thirty-one patients (26.1%) reported relieving of headache from medicine, including ibuprofen, triptans, nimodipine, and some Chinese patent drugs, while 78 patients (65.5%) reported that their headaches were likely to abate after sleep or rest. Thirty-three patients (27.7%) had a symptom of eyeball pain. Headache intensity was rated as 5.9 ± 2.0 (median: 6; range: 1–10) on a visual analog scale (VAS) ranging from 0 to 10 (0 = no pain, 10 = worst imaginable pain). Headache frequency was 16.3 ± 12.5 days (median: 12; range: 1–30) per month.

### Dizziness in HAMD

In our study, 72 patients (60.5%) had a symptom of dizziness, and 19 of them (26.4%) met the diagnose of vestibular migraine according to the appendix criteria in the third edition of the International Classification of Headache Disorders (ICHD3) (12).

### Effect of Revascularization Surgery

Ninety-six patients received extra-intracranial bypass surgery; among them, 52 received direct superficial temporal artery (STA)-middle cerebral artery (MCA) surgery combined with encephalopmyosynangiosis (direct bypass + indirect bypass), and 44 received encephalopmyosynangiosis (indirect bypass) only. The method of bypass surgery was decided on by the neurosurgeons according to the patients' condition. Forty-three received revascularization surgery bilaterally while 53 did so unilaterally. Ninety-six patients showed significant improvements in both scores of headache intensity (VAS; *p* < 0.001) and frequency (days/month; *p* < 0.01) after bypass intervention, but one patient experienced an exacerbation of headache intensity (three preoperatively to five postoperatively in VAS,). These changes were not associated with the headache subtype, sides of surgery (unilateral or bilateral), types of surgical revascularization (direct bypass + indirect bypass or indirect bypass only), or gender (Tables 3–7). There is no difference between the effect of surgical revascularization and conservative treatment on HAMD in a long-term follow-up (*p* > 0.1; Table 8).

**Table 3:** Intensity and frequency of headaches divided by headache subtypes with changes in the frequency (Δ days) and intensity of headaches (Δ VAS) in group of surgical intervention after a long-term follow-up.

	**M**	**TTH**	**M+TTH**	***P*-value**	**95% CI**
**Total** ***n*** **=** **96**					
Δdays	11.97 ± 12.18	15.39 ± 12.32	15.30 ± 12.86	0.445	11.55–16.60
	36	23	37		
ΔVAS	3.66 ± 2.70	3.57 ± 2.76	4.26 ± 2.71	0.541	3.32–4.42

**Table 4:** Divided by age (>17y or ≤17y) with changes in the frequency (Δ days) and intensity of headaches (Δ VAS) in group of surgical intervention after a long-term follow-up.

	**Age>17y**	**Age≤17y**	***P*-value**	**95% CI**
**Total** ***n*** **=** **96**				
*n*	74	22		
Δdays	14.62 ± 12.49	12.22 ± 12.43	0.431	11.55–16.60
ΔVAS	3.51 ± 2.68	5.09 ± 2.49	0.015	3.32–4.42

**Table 5:** Divided by sex with changes in the frequency (Δ days) and intensity of headaches (Δ VAS) in group of surgical intervention after a long-term follow-up.

	**Female**	**Male**	***P*-value**	**95% CI**
**Total** ***n*** **=** **96**				
*n*	62	34		
Δdays	13.35 ± 12.23	15.38 ± 12.94	0.448	11.55–16.60
ΔVAS	3.57 ± 2.59	4.41 ± 2.88	0.148	3.32–4.42

**Table 6:** Intensity and frequency of headaches divided by different type of surgical revascularization(direct bypass+indirect bypass or indirect bypass) with changes in the frequency (Δ days) and intensity of headaches (Δ VAS) after revascularization surgery.

	**Direct bypass + indirect bypass**	**Indirect bypass**	***P*-value**	**95% CI**
**Total** ***n*** **=** **96**				
*n*	52	44		
Δdays	14.77 ± 12.74	13.25 ± 12.21	0.554	11.55–16.60
ΔVAS	3.66 ± 2.76	4.11 ± 2.66	0.420	3.32–4.42

**Table 7:** Intensity and frequency of headaches divided by the lateral of surgical revascularization with changes in the frequency (Δ days) and intensity of headaches (Δ VAS) after revascularization surgery.

	**Unilateral bypass**	**Bilateral bypass**	***P*-value**	**95% CI**
**Total** ***n*** **=** **96**				
*n*	56	40		
Δdays	13.50 ± 12.31	14.88 ± 12.76	0.596	11.55–16.60
ΔVAS	3.46 ± 2.66	4.44 ± 2.72	0.083	3.32–4.42

**Table 8:** Division by surgical intervention/conservative treatment with changes in the frequency (Δ days) and intensity of headaches (Δ VAS) after a long-term follow-up.

	**Surgical intervention**	**Non-surgical treatment**	***P*-value**	**95% CI**
**Total** ***n*** **=** **119**				
*n*	96	23		
Δdays	14.07 ± 12.45	14.52 ± 9.94	0.873	−5.97–5.07
ΔVAS	3.87 ± 2.71	3.74 ± 3.06	0.840	−1.14–1.41

### The Natural Course of HAMD

In our study, 23 patients received conservative treatment which was established on a basis of shared decision-making between physicians and patients and their family members. Conservative treatment indicated a lack of bypass surgery, and no specific medication was recommended. We described the profile of the natural course of HAMD. The twenty-three patients were diagnosed with MMD bilaterally, and none of them were diagnosed with intracranial aneurysm. Among them, 11 had migraine-like headaches, six had tension-type-like headaches, and six had both migraine-like and tension-type-like headaches. The mean age was 34.4 years (median: 37.0 years; SD: 15.5 years; range: 8–71 years). Among them, six were affected by TIA, three had a history of cerebral infarction, two had a history of hemorrhagic stroke, and the other 12 patients had no stroke. The mean follow-up time was 4.9 years (median: 5.0 years; SD: 2.3 years; range: 0.5–10 years). The median duration of headache was 5.5 years (median: 2.0 years; SD: 7.5 years; range: 0.25–30 years). Within 3 months before enrolled, headache intensity was rated 5.9 ± 2.0 (median: 6; range: 3–9) on VAS. Headache frequency was 16.3 ± 12.5 days (median: 12; range: 1–30) per month. As a result of follow-up in the past 3 months, headache intensity was rated 2.0 ± 2.3 (median: 0; range: 0–6) on VAS. Headache frequency was 2.2 ± 5.0days (median: 0; range: 0–30) per month. Twelve patients (52.2%) reported the disappearance of headaches (both intensity and frequency of headache) in the follow-up, and the rest of them (47.8%) reported improvement of both intensity and frequency of headache.

## Discussion

In our study, the majority of these patients (75.5%) reported migraine-like headaches either alone or accompanied by a tension-type-like headache, and this finding is supported by other studies (5). There were no differences regarding the frequency of headache (days/month, *p* > 0.1) before admission, but the intensity of the headache before admission varied (AVS, p = 0.028). We firstly reported that dizziness was one of the frequent symptoms (60.5%) in the patients with MMD-associated headaches; moreover, we did not find any correlations between the presence of dizziness and subtypes of the headache (p > 0.1). Nineteen (26.4%) patients with HAMD and dizziness met the diagnosis of vestibular migraine. Vestibular migraine may be associated with MMD lesions involving the posterior circulation.

The baseline intensity and frequency of the headache of the study patients were significantly severer than the Caucasians (VAS: 5.9 ± 2.0 vs. 3.2 ± 1.3; NRS;16.3 ± 12.5 vs. 6.2 ± 7.8 days/month. There may be several reasons: firstly, the baseline headache frequency calculation adopts 3 months prior to admission, while Kraemer et al. used the annual average headache frequency; secondly, that headache characteristics of Chinese and Caucasian MMD have certain essential differences. Up to now, the pathophysiological mechanisms of HAMD has remained unclear (4, 5, 10, 13–15). The progressive course of steno-occlusion of intracranial vasculature in MMD leads to intracranial blood hypoperfusion, which leads to hypoxia and microvascular ischemia (5, 13). Chronic intracranial hypoxia might be one of the possible mechanisms of primary migraine with aura, which can lead to HAMD (5, 14). Cortical spreading depression triggered by microvascular ischemia might relate to the high prevalence of migraine-like headaches with aura in our study as well as that of Kraemer et al. (5). Dilated leptomeningeal collaterals may be a cause of headaches by stimulation of dural nociceptors (13). Furthermore, some patients may suffer from primary headaches, long-term mental stress of this potentially fatal disease, as well as cognitive impairment of MMD, and these factors may also contribute to the mechanism of HAMD (5, 16–18).

In our study, revascularization surgery greatly improved both of headache frequency and intensity. This is consistent with results from pediatric MMD and adult MMD (5, 7, 8). In the long-term follow-up, there was no difference in headache frequency changes or intensity changes after surgery between patients with different headaches subtypes, headache frequency changes, or intensity changes after surgery and patients with different sides of surgery; there were no differences in headache frequency changes or intensity changes after surgery between patients with different types of bypass surgery. For the long-term outcome of HAMD in our cohort, the conservative treatment group exhibited no different in headache frequency changes or intensity changes compared with the surgical bypass group. These outcomes indicated that the effect of the surgical bypass of HAMD may be a placebo effect. However, the fact should be noted that surgical bypass does decrease the risk of stroke and poor functional outcome, and the improvement of headache frequency and intensity after the surgical bypass was faster than conservative treatment (4, 5, 19–21).

Reduction or disappearance of HAMD (including intensity and frequency of headache) of 23 patients treated conservatively in a long-term follow-up might be related to spontaneous compensation of collateral circulation and redistribution of blood flow in MMD as well as an adaptation of brain tissue or intracranial pain receptors to ischemia and hypoxia; in other words, headache is a part of the symptoms of the process of MMD, and it has the possibility of spontaneous remission or disappearance. Furthermore, patients received conservative treatment may have a less severe phenotype of the disease (with better hemodynamics and lower risk of stroke) compared to those who underwent surgery.

In terms of limitations, our samples all come from a single center, although it is one of the biggest stroke centers in China. Furthermore, HAMD as an isolated symptom was not adequately analyzed in conjunction with hemodynamic data in our study.

The strengths of our study are the size of the cohort, long-term follow-up, and the systematic characterization of HAMD according to the Headache Classification Committee of the International Headache Society (IHS) and The International Classification of Headache Disorders, 2nd edition(ICHD-2) (11). It is a prospective cohort study, and it firstly characterized clinical symptoms of HAMD in the Chinese population. Also, we firstly compared the prognosis of headache in the surgical intervention group and the non-surgery group in a long-term follow-up.

## Conclusion

Migraine-like headache either alone or accompanied with tension-type-like headache were common in patients with HAMD. Dizziness was frequently present with HAMD. There is no difference between the effect of surgical bypass and effect of conservative treatment on HAMD in a long-term follow-up, which indicated that the effect of bypass surgery on HAMD may be a placebo effect. Future research should focus on screening effective drug treatments for HAMD.

## Data Availability Statement

The raw data supporting the conclusions of this article will be made available by the authors, without undue reservation.

## Ethics Statement

The studies involving human participants were reviewed and approved by Beijing Tiantan Hospital Institutional Review Board. Written informed consent to participate in this study was provided by the participants' legal guardian/next of kin.

## Author Contributions

BG: designed the study and drafted the manuscript. KK and JZ: major role in data acquisition and revised the manuscript for intellectual content. DZ and XZ: designed and conceptualized the study, analyzed the data, and drafted and revised the manuscript.

## Conflict of Interest

The authors declare that the research was conducted in the absence of any commercial or financial relationships that could be construed as a potential conflict of interest.
