# Supplementary material for: Clinical Characteristics and Long-Term Outcome of Headaches Associated With Moyamoya Disease in the Chinese Population—A Cohort Study
Source: Front Neurol. 2020 Nov 26;11:605636. doi: 10.3389/fneur.2020.605636 (PMC7726238; doi:10.3389/fneur.2020.605636)
Supplement: Supplementary file 1 [file Data_Sheet_1.PDF]

Headache of MMD patient registration form

Registration Instructions: 1. Please tick " √ " at "☐" to indicate selection;

2. Please fill in words and/or figures in "".

Basic Information:

N a m e		G e n d e r		Years of age		Nationalists	

Prehistory of stroke:

Stroke:Yes ☐ No ☐ if "yes",please indicate the time of onset:

TIA: Yes ☐ No ☐

Cerebral infarction: Yes ☐ No ☐

ICH or SAH: Yes ☐ No ☐

Disease related history:

History of recurrent abdominal pain in childhood: Yes ☐ No ☐ History of recurrent vomiting in childhood: Yes ☐ No ☐

Recurrent episodes of dizziness/dizziness in childhood: Yes ☐ No ☐ History of recurrent torticollis in childhood: Yes ☐ No ☐

Family history of headache: Yes ☐ No ☐

History of present illness:

Headache history: Headache: (months) Aggravation: (months)

Incidence: acute onset ☐ chronic onset ☐ subacute onset ☐

Frequency of attacks: Average (frequency/month) in the past three months

Duration of headache: the average duration of headache in the past three months (days/months)

Duration of each headache (including sleep duration during onset) : not medicated (h);medicated (h)

Headache site: (please choose only) : unilateral ☐ bilateral ☐ not fixed ☐

(optional) : forehead ☐ temporal ☐ occipital ☐ Top ☐ periorbital ☐

Whole head ☐ other ☐

Nature of headache (optional) :

1. Distending pain, dull pain, tension like pain ☐
2. Throbbing pain, jumping pain ☐
3. Tingling, electric shock like pain, tearing like pain ☐
4. Explosive pain ☐
5. Feeling of wearing a hat, pressure like pain, tight collar like pain. Other ☐

VAS score:

The first kind of headache 0 -- 10 (no ,mild,moderate,severe)

Nature of headache:

The second headache 0 -- 10 (no ,mild,moderate,severe)

Nature of headache:

Whether the headache gets worse after fatigue: Yes ☐ No ☐

Headache accompanied by symptoms: nausea ☐ vomiting ☐ photophobia ☐ fear of sound ☐

Whether the headache has aura: Yes ☐ No ☐

Aura symptoms :(optional)

fully reversible motor weakness ☐

fully reversible visual symptoms including positive features (eg, flickering lights, spots or lines) and/or negative features (ie, loss of vision) ☐

fully reversible sensory symptoms including positive features (ie, pins and needles) and/or negative features (ie, numbness)☐

fully reversible dysphasic speech disturbance ☐

Fully reversible symptoms of brain stem: dysarthria ☐ vertigo ☐ tinnitus ☐ Hearing loss ☐ diplopia ☐visual symptoms simultaneously in both temporal and nasal fields of both eyes ☐ Ataxia ☐decreased level of consciousness ☐simultaneously bilateral paraesthesias ☐

Aura characteristics:

Loss or blurring of central vision and/or unilateral sensory symptoms :Yes □ No □

Aura occurrence more than 2 times: Yes □ No □

Whether aura can be fully recovered: Yes □ No □

At least one aura symptom develops gradually over ⩾5 minutes and/or different aura symptoms occur in succession over ⩾5 minutes:Yes □ No □

Each symptom lasts ⩾5 and ⩽60 minutes： Yes □ No □

Headache begins during the aura or follows aura within 60 minutes:Yes □ No □

Aura occurs before headache. ☐ **Headache period.** ☐ **Duration: (minutes)**

Relationship between aura and headache: aura precedes every headache. ☐

Only headache without warning ☐

Individual aura occurrence ☐

Other accompanying symptoms of headache:

Awe - smell ☐ Conjunctival congestion ☐ streaming ☐ nose ☐ running ☐ Forehead and facial sweat ☐

Ptosis ☐ Neck pain ☐ dizziness ☐ Facial pain ☐ Skin tenderness ☐ Pericranial tenderness ☐

Other neurological symptoms (visual impairment ☐ consciousness ☐ body weakness ☐ Body sensation ☐

Psychotic symptoms ☐ epilepsy ☐ Others ☐)

Headache prodrome: fatigue ☐ **difficulty in concentration** ☐ **sensitivity to light and/or sound** ☐ **nausea** ☐

Stiff neck or discomfort ☐ Blurred vision ☐ yawn ☐ pale ☐ Other ☐

Later symptoms of headache: fatigue ☐ **difficulty in concentration** ☐ **Stiff neck** ☐ **Other** ☐

Headache inducing factors: diet ☐ **drinking** ☐ **fatigue** ☐ **sun** ☐ **Weather** ☐ **mood** ☐

Sleep disorder ☐ cough ☐ stoop ☐

Cold stimulation or ingestion or inhalation of cold stimulants ☐ Other ☐

Headache relief factors: rest, sleep ☐ **medication** ☐ **Other** ☐

Emotional state during headache:

Low mood and decreased interest: Yes ☐ no ☐

Tired: Yes ☐ no ☐

Fidgetiness: Yes ☐ No ☐

Emotional fluctuations, the above two situations occur alternately: Yes☐ (frequency:); No☐

Whether there is dizziness during the onset of headache: Yes ☐ No ☐

Dizziness attack situation: dizziness (shaking feeling) ☐ vertigo (rotation feeling) ☐ Dizziness (not clear) ☐

Dizziness attack position: standing ☐ sitting ☐ lying ☐

Fear of heights: Yes ☐ No ☐

Sleep: difficult to fall asleep ☐ sleep is not stable, easy to wake ☐ more sleep ☐ too much ☐

Previous medication: Yes ☐ No ☐ if "yes",please indicate the history of medication:
